# Metabolic stress-like condition can be induced by prolonged strenuous exercise in athletes

**DOI:** 10.1080/03009730802579778

**Published:** 2009-02-04

**Authors:** Stefan Branth, Leif Hambraeus, Karin Piehl-Aulin, Birgitta Essén-Gustavsson, Torbjörn Åkerfeldt, Roger Olsson, Mats Stridsberg, Gunnar Ronquist

**Affiliations:** ^1^Department of Medical Sciences, Clinical Chemistry, University of UppsalaUppsalaSweden; ^2^Department of Biosciences and Nutrition, Karolinska InstitutetHuddingeSweden; ^3^Department of Caring Science, Division for Biomedicine, University of ÖrebroÖrebroSweden; ^4^Department of Clinical Sciences, Faculty of Veterinary Medicine and Animal Science, Swedish University of Agricultural SciencesUppsalaSweden; ^5^Department of Public Health and Caring Sciences/Clinical Nutrition and Metabolism, University of UppsalaUppsalaSweden

**Keywords:** Cellular swelling, energy expenditure, lipid peroxidation, metabolism, myocytes, taurine efflux

## Abstract

Few studies have examined energy metabolism during prolonged, strenuous exercise. We wanted therefore to investigate energy metabolic consequences of a prolonged period of continuous strenuous work with very high energy expenditure. Twelve endurance-trained athletes (6 males and 6 females) were recruited. They performed a 7-h bike race on high work-load intensity. Physiological, biochemical, endocrinological, and anthropometric muscular compartment variables were monitored before, during, and after the race. The energy expenditure was high, being 5557 kcal. Work-load intensity (% of VO_2_ peak) was higher in females (77.7%) than in men (69.9%). Muscular glycogen utilization was pronounced, especially in type I fibres (>90%). Additionally, muscular triglyceride lipolysis was considerably accelerated. Plasma glucose levels were increased concomitantly with an unchanged serum insulin concentration which might reflect an insulin resistance state in addition to proteolytic glyconeogenesis. Increased reactive oxygen species (malondialdehyde (MDA)) were additional signs of metabolic stress. MDA levels correlated with glycogen utilization rate. A relative deficiency of energy substrate on a cellular level was indicated by increased intracellular water of the leg muscle concomitantly with increased extracellular levels of the osmoregulatory amino acid taurine. A kindred nature of a presumed insulin-resistant state with less intracellular availability of glucose for erythrocytes was also indicated by the findings of decreased MCV together with increased MCHC (haemoconcentration) after the race. This strenuous energy-demanding work created a metabolic stress-like condition including signs of insulin resistance and deteriorated intracellular glucose availability leading to compromised fuelling of ion pumps, culminating in a disturbed cellular osmoregulation indicated by taurine efflux and cellular swelling.

## Introduction

Prolonged exercise at a high intensity level elicits marked changes in the metabolic and hormonal milieu (1,2) which can cause a situation of metabolic stress that in turn influences numerous physiological systems, whereas some other responses do not appear to be productive (3). The ultimate outcome of prolonged exercise and physical stress is the effectiveness of the body in converting energy from nutrients into external work and ability to avoid negative impact of metabolic stress caused by high energy turn-over and depleted energy stores resulting in negative energy balance. High-intensity exercise can set various tissues and organs under metabolic stress, especially if substrate needs are not fulfilled and this metabolic regulation involves e.g. important hormonal and immunological responses (4). Hence, exerting physical activity on a very high level for a prolonged time means an energy metabolism that involves many other mechanisms including increased oxidative stress, heat production, activation of inflammatory mediators, and the consequences of cellular dysfunction due to insufficient supply of ATP for sustenance of basic cellular functions like fuelling ion pumps and maintaining cellular volume regulation. The high concentration of the sulphonated amino acid taurine in human muscles suggests an important physiological role in muscle metabolism and function (5).

Few studies have examined energy metabolism during prolonged intense exercise lasting longer than 3 hours (h) (6–8). Studies demonstrate an unequivocal depletion of both glycogen and triglycerides in skeletal muscles subjected to prolonged intensive endurance exercise (9). Moreover, the glycogen depletion pattern is governed by different fibre types (10). Skeletal muscle type I fibres contain higher amount of taurine compared to type II and IIB fibres, and after aerobic endurance events (like marathon) distinct increases in plasma concentrations of taurine have been shown, indicating intracellular muscle cell losses during high-energy demands (5,11).

We wanted to investigate energy metabolic consequences of a prolonged period of continuous strenuous work with very high energy expenditure. Performed work-load can be determined rather exactly during cycling. A standardized aerobic exercise protocol was considered as a transitory condition of negative energy balance eliciting a situation of metabolic stress response in order to compensate for deficient energy supply.

We recruited 12 endurance-trained athletes with a presumed high cycling efficiency and performed a prolonged, high-intensity exercise test during 7 h under standardized and controlled conditions to investigate energy metabolic events on different levels (whole-body level, organ/tissue level, cellular level, and molecular level).

## Materials and methods

### Subjects

Twelve mountain bike cyclists, 6 women and 6 men, all belonging to the Swedish national team, participated in the study. All were on a high and stable training level and considered to be in energy balance, documented by their stable body weight during 2 months preceding the study. Three of the females were on oral contraceptives. Two other females had amenorrhoea. Table I shows physical characteristics of the participants. Basal metabolic rate was calculated according to the equation given by World Health Organization, Energy and Protein Requirements, FAO/WHO/UNU; Geneva, 1985, and by cardiopulmonary indirect calorimetric measurement as described below.

**Table I.** Physical and physiological characteristics of the subjects at base-line.

	All (*n*=12)	Females (*n*=6)	Males (*n*=6)
Age, yr	24.1±3.9	24.8±3.9	23.3±4.1
Height, m	1.74±8.6	1.68±2.7	1.81±8.0 ^a^
BM, kg	66.1±7.7	60.6±3.3	71.6±6.8 ^a^
BMI, kg·m^−2^	21.7±1.2	21.4±1.3	21.9±1.4
Body fat,%	16.1±6.1	21.6±2.7	10.6±1.6 ^a^
LBM, kg	55.6±9.4	46.9±2.2	63.9±5.4 ^a^
BMR, kcal·24 hrs^−1^			
Calculated ^b^	1587±206	1403±47	1770 ±102 ^a^
Measured ^c^	1715±189	1574±102	1857 ±142 ^a^
BMR body weight related, kcal·24 hrs^−1^·kg^−1^			
Total BM	26.0±1.3	26.0±1.6	26.0±1.2
LBM	31.1±2.9	33.2±2.5	29.1±1.2
RER, ratio			
Rest	0.75±0.06	0.72±0.03	0.78±0.08
Work peak	1.11±0.04	1.14±0.04	1.10±0.02
VO_2_ peak			
L·min^−1^	4.5±0.9	3.6±0.1	5.4±0.4 ^a^
ML·kg BM^−1^·min^−1^	67.8±8.3	60.3±1.3	75.3±3.8 ^a^
ML·kg LBM^−1^·min^−1^	80.6±4.9	76.9±2.7	84.2±3.8 ^a^
Work peak heart rate, beats·min^−1^	184±8	183±9	185±6

Values are means±SD; n = 6 females and 6 males. The Wilcoxon rank sum test was used for unpaired comparisons between gender groups: ^a^ *P*<0.05, significantly different from females. ^b^ WHO 1985. ^c^ Indirect calorimetry. BM = body mass; BMI = body mass index; LBM = lean body mass; BMR = basal metabolic rate; RER = respiratory exchange ratio; VO_2_ _peak_=peak oxygen uptake.

All participants were fully informed about the protocol by oral and written information and had given their informed written consent. The study was carried out in accordance with the Declaration of Helsinki II and was approved by the local Ethics Committee at the University Hospital of Uppsala, Sweden.

### Dietary control

Dietary intake for all participants was weighed and registered with an accuracy of 1 g during a preceding 7-day period in 10 of the 12 participants (4 males and 6 females). A computer program (Dietist™, v. 1996, Stockholm, Sweden) was used to calculate all recorded energy and nutrient intake. All participants had similar dietary characteristics, preferentially based on carbohydrate-rich food as typical for endurance-training athletes (65–70 energy% (E%) from carbohydrates, 25–30 E% from fat, and 10–15 E% from protein).

### Experimental design

The study was performed during two consecutive days (day 1 and day 2) during a regular training period. The subjects were told to consume their normal diets prior to the tests. They continued their normal training programme and did not perform any extra vigorous exercise 2 weeks before the tests. There was no training the day before the test except for the VO_2_ peak test.

*Day 1*. In the morning, after an overnight fast and voiding urine, body mass (BM) was registered, and body composition was analysed by means of bioimpedance and skin calliper. Then, an ordinary breakfast was consumed, and all subjects rested for at least 2 h. VO_2_ peak, respiratory exchange ratio (RER), and heart rate (HR) were measured during cycling on a treadmill, using a cardiopulmonary exercise system (Medical Graphics System, CPX2) and a heart frequency monitor system (Polar® Electro, X-trainer™, Finland), respectively.

*Day 2*. In the morning, after an overnight fast, basal metabolic rate (BMR) and RER were measured for 45 min by indirect calorimetry using an ergospirometer (SensorMedics 2900Z, Anaheim, CA, USA), while the subjects were lying awake in bed. Both systems for VO_2_ peak analyses were calibrated between the individual tests by using two known gas mixtures. Thereafter, the subjects had an ordinary breakfast, 2 h prior to the test race.

During exercise, water consumption (including 1 L conventional sports drink, 5% glucose) was *ad libitum*, and the only solid food eaten was bananas. All food and liquid intakes were weighed and recorded during the whole day (including the race) for later calculation.

### Layout of test race

The team coach designed a 200-km race on ordinary paved roads. The start and finish of the race were arranged in connection with the laboratory. The cyclists were told to keep the same velocity, controlled by continuous registration of speed (30 km/h), mechanical power output and cadence, i.e. pedalling rate of around 80 rpm, in order to make the subjects perform the same absolute total work-load. On two occasions during biking, after 50 km (100 min) and 150 km (300 min), expired air was collected in Douglas bags kept in a car driving beside the cyclist. HR was recorded continuously every 5 seconds throughout the whole race using the heart frequency monitoring system.

### Blood sampling and muscle biopsies

Before the start (resting) and immediately after the race, blood samples were collected from a cubital vein using Vacutainer® tubes (two heparin blood tubes, three EDTA blood tubes, one citrate blood tube, and three blood tubes with no anticoagulant)*.* Citrate, EDTA, and heparin blood were cooled immediately in ice water, centrifuged (at 1800 *g*) for 7–8 minutes (min), and then plasma was frozen and kept at 70°C until analysed. Muscle biopsies using the semi-open muscle biopsy technique, were obtained from the vastus lateralis muscle during local anaesthesia before (at rest) as well as within 5 min after termination of the race. The biopsies were taken from the same leg before and after the race with the first distal to the second, 1 cm apart and at the same depth. The biopsies were divided into two parts: one was immediately frozen in liquid nitrogen and stored for later determination of muscle cell triglyceride (MCTG) and glycogen, and the other part was trimmed, mounted, and frozen within 2–3 min in isopentane cooled to the temperature of liquid nitrogen and stored at -80°C until analysis by histochemical methods.

### VO_2_ peak assessment

The VO_2_ peak test was carried out using a regular incremental progressive heightened treadmill exercise protocol cycling to volitional exhaustion. VO_2_, minute ventilation, VCO_2_, and RER were recorded continuously with an automated open-circuit gas analysis system using O_2_ and CO_2_ analysers (Medical Graphics System, CPX2-Spiropharma). HR was recorded simultaneously every 5 seconds using the heart frequency monitor system. VO_2_ and HR peak values at exhaustion were determined when a maximal plateau in VO_2_ was reached despite increased work-load.

### Energy expenditure during exercise

The energy expenditure (EE) was assessed according to calculations using the subjects’ mean HR measurements that were continuously monitored throughout the race and the HR data from the testing of VO_2_ peak from day 1, employing the relation between HR and O_2_ consumption during standardized and maximal cycle work in the laboratory. The mixed expired air that was collected during the race in Douglas bags after 100 and 300 min of exercise was analysed by Beckman OM-14 and Leybold-Heraeus instrumentation for determination of VO_2_ and VCO_2_ and calculation of RER. In addition to obtaining the RER data, these pulmonary O_2_ uptake and expired CO_2_ results were applied to validate the accuracy of using HR monitoring system for the EE and work-load calculations.

### Muscle biopsies

*Biochemical analysis*. The muscle biopsy samples were freeze-dried and dissected free from blood, connective tissue, and debris under a microscope. The muscle biopsy (1–2 mg) intended for glycogen determination was hydrolysed by boiling for 2 h in 1 mol·L^−1^ HCl, and the glucose residues were determined fluorometrically (12). MCTG were analysed by extracting the muscle tissue (1–2 mg) with chloroform-methanol (Folch extract), and the glycerol content was determined enzymatically after evaporation and ester-hydrolysis of the organic solvent phase (13).

*Histochemical analysis*. The biopsy sample obtained for histochemical analysis was cut (10 µm) in a cryostat at −20°C. Transverse serial sections were stained for myosin ATPase after both acid (pH 4.3–4.6) and alkaline (pH 10.3) preincubations. Muscle fibres were identified as type I, IIA, IIB, and IIC, and fibre type composition was determined by examining around 200 fibres per biopsy. The glycogen depletion pattern in the different fibre types was determined based on histochemical PAS staining (14). The fibres were classified as having high, medium, or low staining intensity. Fibre type distribution, fibre areas for each type, relative fibre type distribution, and glycogen depletion pattern were investigated by a computerized image analysis system (Bio-Rad Scan Beam, Hadsund, Denmark), linked to an optical microscope (Leiz, Germany) by a video camera (DAGE MTI, CCD-72).

### Blood analyses

Routine methods at the Department of Clinical Chemistry of the University Hospital of Uppsala were used to analyse blood samples for haematological variables including haemoglobin (Hb), haematocrit (Hct), MCV, MCHC, and serum/plasma concentrations of triglycerides (TG), glucose, creatine kinase (CK), and lactate. Serum concentrations of free fatty acids (FFA) and glycerol were measured by enzymatic colorimetric methods (FFA: Wako Chemical GmbH; Glycerol: Boehringer Mannheim) applied for use in a Monarch 2000 centrifugal analyser. Serum concentrations of insulin were measured by an automated system for immunological analyses (Auto-Delfia, Wallac OY, Turku, Finland). Plasma concentrations of glucagon and leptin were measured using commercial RIA kits (Linco Research Inc., St. Charles, MI, USA). Measurement of IL-6 concentration was performed in citrate plasma with a commercial enzyme-linked immunosorbent assay (ELISA) kit (Quantikine from R&D systems, Abingdon, UK; Enzygnost TAT, Behring Diagnostics, Marburg, Germany). Taurine concentration was analysed in heparin plasma after deproteinization within 10 min by means of 50 mg sulphosalicylic acid/mL plasma and centrifuged and frozen at -70°C. The amino acid level of taurine was thereafter determined by means of an automated amino acid analyser (LKB4151 Alpha PlusAmino Acid Analyser, Pharmacia-LKB Biochrom, Cambridge, UK) by use of a lithium buffer system according to the manual. Each analysis had DL-2,4-diamino-*n*-butyric acid as internal standard (15). Malondialdehyde (MDA) was analysed in EDTA plasma, by using an HPLC fluorimetric detection method (16).

### Body composition measurements

BM was determined using high-precision scales (type KC120-ID 1 Multirange Mettler Instruments, Greifenesee, Switzerland) with a precision of ±0.05 kg. *Skinfold thickness* was measured in four locations on the right side of the body (biceps, triceps, subscapular, and suprailiac folds, respectively) using Harpenden skin calliper (John Bull, British Indicators, St. Albans, England). *Bioimpedance spectroscopy* (BIS) measurements that used multiple frequencies from 5 kHz to 1000 kHz were performed using XITRON 4200 (Xitron Technologies Inc., San Diego, CA, USA). Total body composition data were calculated by means of the three-compartment model described by Forslund et al. (17). Leg segment volume was determined by water displacement volumetry (18). Prior to these measurements, the length and location of the leg segment were determined by specifically made equipment that measures the distance from the foot to the lower part of the pelvis. Based on this measurement the upper and lower limit of the leg segment was established and marked carefully on the legs (near the great trochanter and ankle, respectively). In two in-house-developed water tanks the total leg volume and foot volume were measured on the right side. Leg segment volume was calculated by subtracting foot volume from total leg volume. The water tanks were filled with water (30°C). Before and after exercise the right leg was carefully placed into a water-filled tank. The equilibrated over-flowed water was collected and weighed on the high-precision scales, and water volume (that equals leg volume) was calculated. Before exercise the right foot volume was quantified in a foot water tank. The remaining water in the foot tank, after the foot had been immersed and removed, was weighted and volume calculated. The same foot volume was used in the post-exercise calculations. The same leg segment marks were used to place the electrodes during segmental BIS measurements. All athletes were assessed lying on a bench by the same experienced investigator. Software provided by Xitron was used to calculate segmental extracellular and intracellular resistance, and, together with segmental volume data derived by water displacement volumetry, the amounts of intra- and extracellular fluids (ICW and ECW) were calculated. Total body fluid in the segment (TBW) was calculated as the sum of ECW and ICW.

### Statistical analyses

The Statistica® software was used for all calculations. Study variables were first reviewed for means and dispersion measures. Differences between the two sampling times were analysed by Student's *t* test for paired samples with regard to variables with normal distributions and with non-parametric methods (Wilcoxon signed rank test) for all other variables. Owing to non-normal distributions and small sample sizes (*n*=6 per group), quantitative variables were compared between groups (genders) using non-parametric methods. The Wilcoxon rank sum test was used for unpaired comparisons between groups, and the Wilcoxon signed rank test was used for paired comparisons (e.g. change over time). Correlations were calculated using the Spearman correlation coefficient. A *P*-value <0.05 was considered significant; no adjustments were made for multiple comparisons. All data presented are means±SD.

## Results

All subjects were in energy balance the days before the study (base-line). The BMR values were significantly lower in the females in absolute terms but no gender-related difference was observed when referred to BM or lean body mass (LBM). The VO_2_ peak was significantly lower in the females in absolute terms (L·min^−1^) as well as per kg BM and LBM (mL·kg^−1^·min^−1^). However, no gender-related differences were observed concerning means of resting and peak HR values, nor concerning RER (Table I).

### Work-load

The race lasted for 416 min in all subjects, according to the schedule. All participants kept the prearranged mean velocity (30 km per h) and the same cadence, with minor non-significant individual differences. The mean HR was 136±10 beats/min during the total work period, significantly higher in females (142±9 beats/min) than in males (129±6 beats/min). This corresponded to a mean work-load intensity of 73.8±5% with a significantly higher relative work-load intensity in females (77.7±4%) than in males (69.9±2%), when expressed as percentage of peak HR.

### Energy expenditure

**Figure 1.**
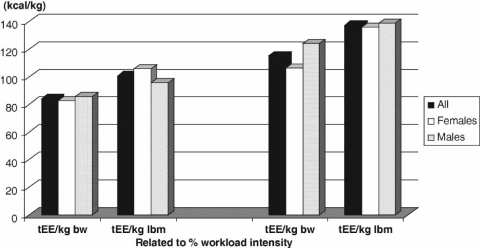
Energy expenditure (EE) normalized to kg body weight (bw) and lean body mass (lbm) and additionally related to per cent work-load intensity (% of VO_2_ peak) during the 7-h race in all subjects (*n*=12), females (*n*=6) and males (*n*=6).

Because of technical error the Douglas bag collections failed in one male and two females. However, the CO_2_ and O_2_ gas analyses from the Douglas bag collections from the nine other subjects were concordant and confirmed the same EE data and individual relative work-load as obtained by their calculations from the HR monitoring system during the race and CO_2_, O_2_, and HR measurements from the VO_2_ peak test. The HR monitoring system was the principal EE test in our study and, hence, the former results agreed with this principal test. The absolute total EE (TEE) during the 7-h race was 5557±763 (mean) kcal, significantly lower in females (5000±370 kcal) than in males (6114±632 kcal) (Table II). However, relatively small differences between sexes were observed when TEE was related to kg BM and to kg LBM as seen in Figure 1. Mean TEE related to kg BM was 84.2±7.5 kcal·kg^−1^, and to kg LBM, 100.7 ±0.2 kcal·kg^−1^. A further adjustment of TEE related to BM and LBM in relation to work-load intensity during the race (percentage of VO_2_ peak) of each subject was performed and showed for the BM-related TEE a mean value of 115.2±2 kcal·kg^−1^ and for the LBM-related TEE a mean value of 137.3±11 kcal·kg^−1^. Least differences between sexes were obtained when TEE was adjusted for by both LBM and work-load intensity (Figure 1).

**Table II.** Work-load, energy expenditure, and respiratory exchange ratio during the 7-h race.

	All (*n*=12)	Females (*n*=6)	Males (*n*=6)
Total EE, kcal	5557±763	5000±370	6114±632 ^a^
RER, ratio			
at rest	0.75±0.06	0.72±0.03	0.78±0.08
at 100 min exercise	0.82±0.03	0.81±0.02	0.83±0.04
at 300 min exercise	0.78±0.03	0.77±0.02	0.79±0.04

Values are means±SD; *n*=6 females and 6 males. The Wilcoxon rank sum test was used for unpaired comparisons between gender groups: ^a^ *P*<0.05, ^b^ *P*<0.01, significantly different from females. EE = energy expenditure; RER = respiratory exchange ratio.

The energy intake from breakfast (900 kcal in females and 1500 kcal in males) and during the race (1170 kcal in females and 870 kcal in males) constituted only 41% and 39% of TEE in females and males, respectively. Accordingly, a highly negative energy balance of about -3334 kcal was achieved during the 7-h race (−2922 kcal in females and −3747 kcal in males).

At rest, RER was 0.75±0.06, and after 100 and 300 min exercise RER showed a slight but non-significant elevation compared to the resting value (0.82±0.03 and 0.78±0.03, respectively).

### Fibre distribution and energy stores in muscle

Muscle fibre distribution, energy stores and their utilization are presented in Tables III and IV. In one case, only fibre type distribution and total glycogen content were analysed due to lack of material. Prior to the race, all participants showed glycogen-loaded muscle fibres. The initial muscle glycogen concentration was on an average 702.1±09 mmol·kg^−1^ dry wt, of which 65.8±14% was utilized, corresponding to a significant glycogenolysis of 460.6±08 mmol·kg^−1^ dry wt during the race. At the end of the race almost all type I fibres (94.5±7%) were depleted of glycogen; 21±27% of the type IIA and 10±12% of the type IIB fibres were glycogen-depleted. No statistically significant differences in muscle glycogen stores, utilization, or (fibre type) glycogen depletion pattern were shown between sexes.

**Table III.** Muscle fibre distribution in vastus lateralis muscle in females and males.

	Females and males		Females		Males	
Fibre type	%/no.	%/area	%/no.	%/area	%/no.	%/area
I	56.1±9	55.9±14	61.4±8	61.0±14	50.8±6	50.8±14
IIA	36.1±6	38.5±12	32.4±5	34.2±14	39.8±5	42.7±10
IIB	6.7±6	5.3±4	4.4±4.3	4.1±3	9.1±8	6.1±4
IIC	1.1±1	0.5±0.7	1.8±1.4	0.6±0.8	0.5±0.5	0.6±0.7

Values are means±SD; *n*=6 females and 6 males. No statistically significant differences were seen between females and males (*P*<0.05).

**Table IV.** Muscular glycogen and triglyceride (TG) content before (resting condition) and after the 7-h race.

	All *n*=12	Females *n*=6	Males *n*=6	*P*-value ♀ versus ♂
Glycogen, mmol/kg dry wt:				
Before race (rest)	702.1±109.1	696.8±129.0	707.3±97.3	ns
After race	241.5±105.1 ^d^	204.8±128.9 ^c^	278.2±66.4 ^d^	ns
Utilization during the race:				
mmol/kg dry wt	460.6±108.0	492.1±113.3	429.2±102.2	ns
mmol/h	66.5±15.6	71.0±16.4	61.9±14.7	ns
%	65.8±14.3	71.4±16.4	60.2±9.8	ns
Triglycerides, mmol/kg dry wt:	*n*=11	*n*=5	*n*=6	
Before race (rest)	18.4±8.2	21.8±10.2	15.0±4.2	ns
After race	7.8±3.7 ^b^	8.8±1.4	7.0±4.9 ^b^	ns
Utilization during the race:				
mmol/kg dry wt	10.1±8.0	12.6±11.0	8.0±4.3	ns
mmol/h	1.5±1.2	1.8±1.6	1.2±0.6	ns
%	53.0±20.8	52.0±19.9	54.0±23.3	ns

Values are means±SD, for 12 subjects as one group put together (All) as well as separated in 6 females and 6 males (muscular glycogen), and 5 females and 6 males (muscular triglycerides). Wilcoxon signed rank test was used for paired comparisons (i.e. for change over time during the race) within each group, ^a^ *P*<0.05; ^b^ *P*<0.01; ^c^ *P*<0.005; ^d^ *P*<0.001. The Wilcoxon rank sum test was used for unpaired comparisons between groups, ♀ versus ♂, *P*-value for difference between genders in the change from rest (before) to the end (after) of the race. No significant differences were seen between the genders in any values neither before (rest) nor after the race.

**Figure 2.**
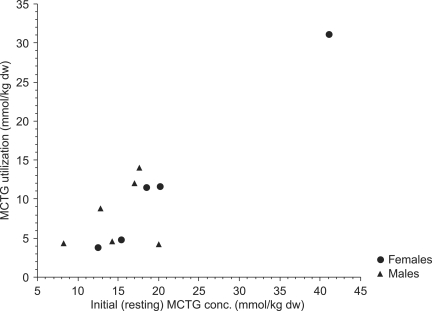
Relationship between initial muscle cell triglyceride (MCTG) content and MCTG utilization during the 7-h race. Correlation was calculated using the Spearman correlation coefficient (*r*=0.61, *P*<0.05, *n*=11).

**Figure 3.**
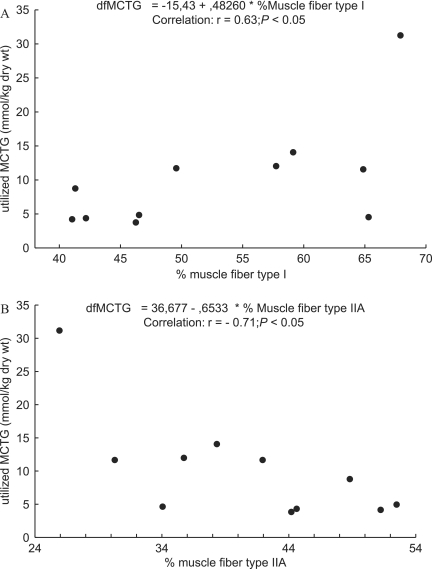
A: Relationship between per cent (%) muscle fibre type I and muscle cell triglyceride (MCTG) utilization during the 7-h race. Correlation was calculated using the Spearman correlation coefficient (*r*=0.63, *P*<0.05, *n*=11). B: Relationship between per cent (%) muscle fibre type IIA and muscle cell triglyceride (MCTG) utilization during the 7-h race. Correlation was calculated using the Spearman correlation coefficient (*r* =-0.71, *P*<0.05, *n*=11).

At rest, the MCTG concentration of the 11 measured subjects was 8.4±8.2 mmol·kg^−1^ dry wt and decreased significantly by, on an average, 10.1±8.0 mmol·kg^−1^ dry wt (53%) during the race. A significant correlation (*r*=0.61, *P*=0.047, *n*=11) was found between the initial MCTG content and its utilization during the race (Figure 2). No significant gender-related differences were noted concerning the MCTG concentration and its utilization. Subdivided into fibre types, an apparent discrepancy was revealed. Hence, type I fibres showed a clear inclination towards lipolysis (*r*=0.63; *P*<0.05) (Figure 3A), while such a disposition was not the case for type IIA fibres, which instead showed an inverse relationship to the MCTG utilization (*r*=-0.71; *P*<0.05) (Figure 3B).

### Haematologic findings

At rest, blood haemoglobin (Hb) and haematocrit (Hct) were within normal ranges, 141.8±7.4 g·L^−1^ and 41.7±2.1%, respectively. After the race, no significant change in Hb was seen, 145.1±7.4 g·L^−1^. Hct was also unchanged after the race, 41.5±2.1%. Blood MCV showed a statistically significant volume decrease from on an average 94.0±2.9 to 90.2±3.0 fL (*P*<0.001), while MCHC concentration increased from 339±5.8 to 351.1±3.3 g·L^−1^ (*P*<0.001). No significant gender-related differences in blood haematological parameters were seen.

**Figure 4.**
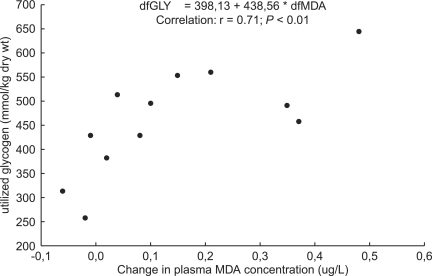
Relationship between change in plasma MDA (malondialdehyde) concentration and glycogen utilization during the 7-h race. Correlation was calculated using the Spearman correlation coefficient (*r*=0.71, *P*<0.01, *n*=12).

*Biochemical responses*. Concentrations of FFA, glycerol, MDA, CK, taurine, and IL-6 in serum and plasma at rest and after the race are shown in Table V. Statistically significant increases were observed in serum concentrations for glycerol, FFA, and CK, and in plasma concentrations for lactate, MDA, taurine, and IL-6. Females had a significantly higher glycerol increase than males. A significant correlation (*r*=0.71, *P*<0.01) was found between the changes in plasma MDA concentration and glycogen utilization (Figure 4).

**Table V.** Some plasma (P)/serum (S) metabolite concentrations before (resting conditions) and after the 7-h race.

	All	Females	Males	*P*-value ♀ versus ♂
P—glucose, mmol·L^−1^				
Before (rest)	5.1±0.3	5.0±0.3	5.2±0.2	
After exercise	5.4±0.4 ^a^	5.4±0.5	5.4±0.3	ns
P—lactate, mmol·L^−1^				
Before (rest)	1.1±0.2	1.1±0.2	1.1±0.2	
After	1.8±0.7 ^c^	1.9±0.9	1.7±0.5 ^b^	ns
S—FFA, mmol·L^−1^				
Before (rest)	0.24±0.1	0.28±0.1	0.20±0.1	
After	1.65±0.4 ^d^	1.84±0.3 ^d^	1.46±0.4 ^d^	ns
S—glycerol, mmol·L^−1^				
Before (rest)	0.09±0.03	0.09±0.02	0.09±0.02	
After	0.43±0.1 ^d^	0.50±0.1 ^d^	0.36±0.1 ^d^	<0.05
P—MDA, µg·L^−1^				
Before (rest)	0.28±0.1	0.35±0.1	0.24±0.1	
After	0.38±0.1 ^a^	0.40±0.1	0.36±0.1	ns
S—CK, ukat·L^−1^ (microkat·L^−1^)				
Before (rest)	1.88±1.0	1.37±0.2	2.40±1.3	
After	3.10±1.1 ^d^	2.62±0.5 ^c^	3.60±1.5 ^c^	ns
P—Taurine, mmol·L^−1^				
Before (rest)	49.6±12.1	51.3±15.9	47.8±7.9	
After	187.4±56.8 ^d^	210.5±59.5 ^d^	164.3±47.7 ^d^	ns
P—IL-6, mmol·L^−1^				
Before (rest)	2.3±4.3	1.3±1.4	3.3±5.6	
After	9.8±5.2 ^d^	11.6±3.8 ^d^	8.0±6.1	ns

Values are means±SD, for 12 subjects, put together as one group (All) and as separated in gender groups, females (*n*=6) and males (*n*=6). Wilcoxon signed rank test was used for paired comparisons (i.e. for change over time during the race) within each group: ^a^ *P*<0.05; ^b^ *P*<0.01; ^c^ *P*<0.005; ^d^ *P*<0.001. The Wilcoxon rank sum test was used for unpaired comparisons between groups, ♀ versus ♂, *P*-value for difference between genders in the change (response) from rest (before) to the end (after) of the race. No significant differences were seen between the genders in any values neither before (rest) nor after the race. P = plasma; S = serum; FFA = free fatty acids; MDA = malondialdehyde; CK = creatine kinase.

### Endocrine responses

Hormonal data are presented in Table VI. The concentrations of serum insulin were unchanged after the 7-h race. Plasma concentration of glucagon increased in all subjects during the race. Serum concentration of leptin decreased in both genders, most distinctly in females who had significantly higher serum levels of leptin than males, both at rest and after the race.

**Table VI.** Plasma (P)/serum (S) hormone concentrations before (resting condition) and after the 7 h race.

	All	Females	Males	*P*-value ♀ versus ♂
S-insulin, mU/L				
Before (rest)	5.2±2.2	4.8±1.7	5.6±2.7	
After	4.8±2.2	4.5±2.0	5.0±2.6	ns
P-Glucagon, ng/L				
Before (rest)	64.1±11	60.0±9	68.2±13	
After	93.2±24 ^d^	91.8±30 ^a^	94.5±20 ^d^	ns
S-leptin, nmol/L				
Before (rest)	4.0±3.3	6.4±3.2	1.6±0.4 ^g^	
After	2.0±0.9 ^a^	2.6±0.8 ^a^	1.3±0.3 ^g^	<0.01

Values are means±SD, for all 12 subjects put together as one group (All) as well as separated in gender groups, females (*n*=6) and males (*n*=6). Wilcoxon signed rank test was used for paired comparisons (i.e. for change over time during the race) within each group: ^a^ *P*<0.05; ^b^ *P*<0.01; ^c^ *P*<0.005; ^d^ *P*<0.001. The Wilcoxon rank sum test was used for unpaired comparisons between groups: ^e^ *P*<0.05; ^f^ *P*<0.01; ^g^ *P*<0.005; males versus females values at rest (before) respectively at end (after) of the race; and ♀ versus ♂, *P*-value for difference between genders in the change (response) from rest (before) to the end (after) of the race. P = plasma; S = serum.

### Leg volume alterations

**Figure 5.**
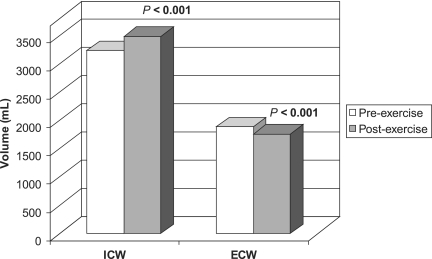
Fluid distribution (mL) in subcompartments of the leg. Intracellular fluids (ICW) and extracellular fluids (ECW) were measured before and after the 7-h race.

The total leg volume, measured before and after exercise, decreased slightly from 7783±1242 to 7602±1150 mL (*P*<0.01). A partitioning into water subcompartments revealed that ICW increased from 3233±767 to 3482±768 mL (*P*<0.001) and ECW decreased from 1884±397 to 1760±329 mL (*P*<0.01). TBW (total body water) changed accordingly from 5117±1142 to 5242±1083 mL. The calculated percentage water content of the whole leg was almost the same, being 65.3±6.5% before and 68.6±6.0% after exercise (*P*=0.11). Per cent ICW of the total water content increased from 63.0±2.2 to 66.2±1.8% (*P*<0.001), whilst per cent ECW of total water content of the leg decreased from 37.0±2.2% to 33.8±1.8% (*P*<0.001) (Figure 5). Accordingly, the ICW/ ECW ratio showed a large increase from 1*.*71±0.16 to 1.97±0.16 (*P*<0.001). Herewith a higher ICW/TBW ratio after the race (0.63±0.02 versus 0.66±0.02; *P*<0.001) was apparent.

## Discussion

The present results show that prolonged strenuous exercise (7 h cycling) performed by well trained athletes under controlled conditions induced pronounced changes in metabolic, endocrinological, and anthropometric muscular compartment variables, culminating in a state of metabolic stress. We observed a very high energy expenditure rate during the race. As expected the local energy stores were utilized to a large extent. To our knowledge, few studies have so far been published dealing with metabolic demands during exercise at such a work-load intensity level for a prolonged time period (19). A majority of studies examining energy metabolism under similar conditions has used a competition as the exercise stimulus, which introduces a number of confounding factors such as mental stress, environmental conditions, and fluctuating intensity (20). Since differences in the environmental conditions, equipment, and technical skills were negligible among the subjects of this study, disparity in sources of substrates must be explained by different efficiency of the metabolic pathways in addition to differences in relative work-load and anthropometry. Although this unusually high amount of absolute work-load was kept similar for all participants, the relative work-load (work-load intensity) was higher in females than in males expressed as a relationship to VO_2_ peak per kg LBM (being 77.7% and 69.9% of the VO_2_ peak in females and males, respectively). This circumstance makes any further direct comparisons between specific substrate utilization of genders more difficult. Since the distance and time of on-going work in physical terms were exactly the same for all athletes, energy cost for the race should be the same. However, activity-induced energy expenditure is also dependent on body size and total cell mass. Thus, although there is no consensus regarding the mode of normalization for body size, it is most likely that the active muscle mass plays the crucial role, irrespective of gender differences in energy expenditure during exercise. However, Zehnder et al. (21) showed recently a higher TEE per kg LBM in males compared with females during a 3-h moderate exercise test in well trained cyclists under similarly standardized conditions. This finding was contrary to our study, where we did not observe any statistically significant gender-related difference in TEE when adjustment for LBM had been performed in agreement with Westerterp's comprehensive considerations in this regard 22. It is interesting to note in this context that, when an additional adjustment was made to the varying work-load intensity, a further improvement of the correction seemed to occur (Figure 1). Accordingly, not only muscle mass but also work-load intensity in itself might be of importance.

Muscle fibre composition influences the performance and energy metabolism during exercise, especially during a prolonged period of strenuous work 10. The glycogen utilization exceeding 93% in both males and females was extraordinary in the type I fibres of the athletes. It has for a long time been generally agreed that gender-related differences exist in the metabolic response during endurance exercise, and most studies on lipolysis during exercise have indicated that females oxidize more fatty acids than males (23–25). This reasoning was corroborated by our finding in that circulating glycerol was increased in females compared to males during exercise, being in agreement with other studies as well (26–28). It should be noted though that the difference between sexes in the release of FFA was not as apparent as that of glycerol. In the light of the fact that when exercise intensity increases, a progressive enhancement takes place in the utilization of carbohydrate sources (29), one would have expected that the females in our study should have had an increased rate of carbohydrate utilization. However, as the glycogen utilization and glucose levels were about the same in both sexes and the females showed signs of enhanced lipolysis, indicated by the higher serum glycerol level, this lipolysis might reflect a possible relative extra energy requirement of the females due to their increased work-load intensity. A strong positive correlation existed between the initial MCTG content and its utilization during exercise (Figure 2), confirmatory of previous studies (24,21). Due to some contradictory results there has been a controversy about the contribution of the MCTG pool as a substrate source to the total energy expenditure during exercise (19). However, it is possible to show a time-dependent decline in MCTG content during more prolonged (3–7 h) exercise at a moderate to high-intensity level, as revealed by a 53% reduction of MCTG in our study. Such a high usage rate has not attracted much attention in the literature (6,7,10). Moreover, the energy utilization of MCTG in relation to LBM correlated positively to per cent of type I fibre area of the subjects and negatively to per cent of type IIA fibre area (Figure 3A and B). This is in accordance with other studies reporting greater lipid content and higher FFA oxidation capacity in the type I fibres (8). Also, our study revealed a very high fatty acid turn-over by the 7-fold increase in FFA and 5-fold increase in glycerol concentrations in serum.

Glycogenolysis concomitantly with a moderately increased lactate concentration in plasma was indicative of a state of metabolic stress, a condition that might involve a certain degree of insulin resistance. Some investigators have also found that blood glucose concentration tends to increase at work-load intensities exceeding 80% of VO_2_ peak in absence of carbohydrate ingestion (30). Most endurance exercise studies (without carbohydrate supplementation and under depleted glycogen store conditions) bear testimony to blood glucose levels being stable or slightly decreased as a sign of the increased dependency on fat oxidation (31). As a consequence, and in accordance with the general opinion, insulin levels decrease during a prolonged exercise session. Our study revealed some contradictory results in that circulating glucose levels were increased concomitantly with an unchanged serum insulin concentration. This somewhat paradoxical situation might reflect an insulin resistance based on metabolic stress on a cellular level and/or increased proteolytic glyconeogenesis (15). A metabolic stress-like state is also supported by the elevated plasma MDA concentration after the race. What is more, the MDA levels correlated to the glycogen utilization rate and thereby also indirectly to energy turn-over rate and/or substrate deficit (Figure 4). It should be kept in mind that muscular contraction increases the production of reactive oxygen species (32), and lack of energy on the cellular level causes oxidative stress which may in concert damage membranous structures by lipid peroxidation.

Moreover, metabolic exhaustion means an imbalance at the cellular level between ATP on one hand and ADP, AMP, and adenylate catabolites, on the other. This in turn leads to less ATP to fuel ion pumps, especially the Na^+^/K^+^ ATPase. The decreased electrochemical potential results in not only increased Na^+^ but also Ca^2+^ and water inside the cell causing cellular swelling, as was noted in the present study regarding leg muscle cells. Accordingly, a distinct intracellular swelling of the leg muscles occurred in all athletes after the race, probably also leading to cellular leakage, verified by e.g. an increased concentration of CK in serum. Cellular swelling is to some extent counteracted by taurine, known as an osmoregulator in many cells (33). The cellular uptake of the amino acid taurine is Na^+^-dependent, and intracellular taurine represents an energy state of the cell (osmotic work has been performed at the expense of the Na^+^ gradient). In any case of cell energy metabolic disturbance, as has been described here, taurine efflux along its gradient (downhill) can occur together with Na^+^ against its gradient (uphill). This extracellular Na^+^ can in turn be utilized in a Na^+^/Ca^2+^ counter-transport, reducing the intracellular Ca^2+^ load, and again, intracellular Na^+^ can undergo co-transport with intracellular taurine out of the cell as described above. The final result is an increased extracellular accumulation of taurine to favour cell survival. This was well reflected by our data. In accordance, other investigators have shown similar increases in plasma taurine concentrations after endurance events such as marathon races (5,11,34), indicating a role in muscular cell stress during energy-demanding muscular work. Moreover, Kavianipour and collaborators (35) have shown that taurine muscle cellular efflux reflects the extent of myocardial ischaemic injury in an experimental animal model.

Human erythrocytes have highly specific insulin receptors with binding characteristics similar to other insulin target sites, e.g. myocytes, hepatocytes, and adipocytes (36). Similarly to liver and fat cells, erythrocytes have high- and low-affinity insulin receptors and contain α-subunits for binding, and β-subunits, i.e. tyrosine kinase, that display sulfhydryl groups for enzymatic activity (37). Increased levels of circulating insulin can cause downregulation of its receptors. A reduced number of insulin receptors have indeed been demonstrated in erythrocytes of both hyperinsulinaemic and non-insulinaemic hypertonic patients (38). We cannot exclude the possibility of downregulated insulin receptors and a reduced glucose uptake into erythrocytes of our study athletes. Hence, it is reasonable to suggest that our subjects displayed a relative deficiency of intracellular glucose in their erythrocytes during the race. Therefore, ATP fuelling the Na^+^/K^+^ ATPase in the erythrocytes might have been deficient. This again leads to a decreased electrochemical potential resulting in increased erythrocyte levels of not only Na^+^ but also Ca^2+^ and water. Increased Ca^2+^ in erythrocytes elicits the opening of the Ca^2+^-sensitive K^+^ channel leading to a selective efflux of K^+^ (Gardos effect) (39). This in turn results in cellular dehydration (xerocytosis) reflected by distinctly diminished MCV values in our athletes concomitantly with distinctly elevated MCHC values (haemoconcentration). In this context it is interesting to note that we found similar relationships concerning athletes (sailors) exposed to strong energy metabolic stress-like conditions. This meant that these athletes also developed an incipient insulin-resistant state (40). In addition, their erythrocytes also displayed cell volume shrinkage, and in the erythrocytes increased Ca^2+^ concentration was indeed demonstrated (unpublished data measured by the PIXE -proton-induced X-ray emission method).

Another indication of a metabolic stress-like condition could be our observation of a manyfold elevation of IL-6 levels after the race. Prolonged exercise causing glycogen depletion is associated with increased levels of certain cytokines (e.g. IL-6) and higher levels of cytokine IL-6 may indicate a higher level of metabolic stress (41). Moreover, the exercise-linked increase of IL-6 and insulin is coupled to the activation of the lipolysis in the adipocytes (42), which in turn leads to a decrease in leptin secretion irrespective of body fat stores. All these IL-6-induced metabolic changes are in line with our present study.

## Conclusions

Twelve endurance-trained athletes were subjected to a 7-h strenuous exercise session leading to unusually high energy expenditure and substrate utilization. Due to these circumstances a metabolic stress-like condition developed including signs of insulin resistance and deteriorated intracellular glucose availability leading to supposedly compromised fuelling of ion pumps, culminating in a disturbed cellular osmoregulation. Taurine might have an important role protecting cells from abnormal accumulation of ions during metabolic cellular stress.

## References

[1] Ahlborg G, Felig P, Hagenfeldt L, Hendler R, Wahren J (1974). Substrate turnover during prolonged exercise in man. Splanchnic and leg metabolism of glucose, free fatty acids, and amino acids. J Clin Invest.

[2] Hartley LH, Mason JW, Hogan RP, Jones LG, Kotchen TA, Mougey EH (1972). Multiple hormonal responses to prolonged exercise in relation to physical training. J Appl Physiol.

[3] Newcomer BR, Sirikul B, Hunter GR, Larson-Meyer E, Bamman M (2005). Exercise over-stress and maximal muscle oxidative metabolism: a 31P magnetic resonance spectroscopy case report. Br J Sports Med.

[4] Coyle EF (2000). Physical activity as a metabolic stressor. Am J Clin Nutr.

[5] Cuisinier C, Ward RJ, Francaux M, Sturbois X, de Witte P (2001). Changes in plasma and urinary taurine and amino acids in runners immediately and 24h after marathon. Amino Acids.

[6] Bergstrom J, Hultman E, Saltin B (1973). Muscle glycogen consumption during cross-country skiing (the Vasa ski race). Int Z Angew Physiol.

[7] Froberg SO, Mossfeldt F (1971). Effect of prolonged strenuous exercise on the concentration of triglycerides, phospholipids and glycogen in muscle of man. Acta Physiol Scand.

[8] van Loon LJ (2004). Use of intramuscular triacylglycerol as a substrate source during exercise in humans. J Appl Physiol.

[9] Jansson E, Kaijser L (1987). Substrate utilization and enzymes in skeletal muscle of extremely endurance-trained men. J Appl Physiol.

[10] Costill DL, Gollnick PD, Jansson ED, Saltin B, Stein EM (1973). Glycogen depletion pattern in human muscle fibres during distance running. Acta Physiol Scand.

[11] Ward RJ, Francaux M, Cuisinier C, Sturbois X, De Witte P (1999). Changes in plasma taurine levels after different endurance events. Amino Acids.

[12] Lowry OH, Passonneau JV (1971). Some recent refinements of quantitative histochemical analysis. Curr Probl Clin Biochem.

[13] Chernick SS (1969). Determination of glycerol and acylglycerol. Methods Enzymol.

[14] Pearse AG (1961). Appendix 9. In: Histochemistry—Theoretical and Applied.

[15] Forslund AH, Hambraeus L, van Beurden H, Holmback U, El-Khoury AE, Hjorth G (2000). Inverse relationship between protein intake and plasma free amino acids in healthy men at physical exercise. Am J Physiol Endocrinol Metab.

[16] Ohrvall M, Tengblad S, Ekstrand B, Siegbahn A, Vessby B (1994). Malondialdehyde concentration in plasma is inversely correlated to the proportion of linoleic acid in serum lipoprotein lipids. Atherosclerosis.

[17] Forslund AH, Johansson AG, Sjödin A, Bryding G, Ljunghall S, Hambraeus L (1996). Evaluation of modified multicompartment models to calculate body composition in healthy males. Am J Clin Nutr.

[18] Brijker F, Heijdra YF, Van Den Elshout FJ, Bosch FH, Folgering HT (2000). Volumetric measurements of peripheral oedema in clinical conditions. Clin Physiol.

[19] Watt MJ, Heigenhauser GJ, Dyck DJ, Spriet LL (2002). Intramuscular triacylglycerol, glycogen and acetyl group metabolism during 4 h of moderate exercise in man. J Physiol.

[20] Meyer T, Gabriel HH, Auracher M, Scharhag J, Kindermann W (2003). Metabolic profile of 4 h cycling in the field with varying amounts of carbohydrate supply. Eur J Appl Physiol.

[21] Zehnder M, Ith M, Kreis R, Saris W, Boutellier U, Boesch C (2005). Gender-specific usage of intramyocellular lipids and glycogen during exercise. Med Sci Sports Exerc.

[22] Westerterp KR, Tarnopolsky M (1985). Nutritional implications of gender differences in metabolism: Energy metabolism, human studies.

[23] Horton TJ, Pagliassotti MJ, Hobbs K, Hill JO (1998). Fuel metabolism in men and women during and after long-duration exercise. J Appl Physiol.

[24] Steffensen CH, Roepstorff C, Madsen M, Kiens B (2002). Myocellular triacylglycerol breakdown in females but not in males during exercise. Am J Physiol Endocrinol Metab.

[25] Tarnopolsky MA, Atkinson SA, Phillips SM, MacDougall JD (1995). Carbohydrate loading and metabolism during exercise in men and women. J Appl Physiol.

[26] Burguera B, Proctor D, Dietz N, Guo Z, Joyner M, Jensen MD (2000). Leg free fatty acid kinetics during exercise in men and women. Am J Physiol Endocrinol Metab.

[27] Carter SL, Rennie C, Tarnopolsky MA (2001). Substrate utilization during endurance exercise in men and women after endurance training. Am J Physiol Endocrinol Metab.

[28] Mittendorfer B, Horowitz JF, Klein S (2002). Effect of gender on lipid kinetics during endurance exercise of moderate intensity in untrained subjects. Am J Physiol Endocrinol Metab.

[29] Kristiansen S, Gade J, Wojtaszewski JF, Kiens B, Richter EA (2000). Glucose uptake is increased in trained vs. untrained muscle during heavy exercise. J Appl Physiol.

[30] Below PR, Mora-Rodriguez R, Gonzalez-Alonso J, Coyle EF (1995). Fluid and carbohydrate ingestion independently improve performance during 1 h of intense exercise. Med Sci Sports Exerc.

[31] Millard-Stafford ML, Sparling PB, Rosskopf LB, DiCarlo LJ (1992). Carbohydrate-electrolyte replacement improves distance running performance in the heat. Med Sci Sports Exerc.

[32] Reid MB, Shoji T, Moody MR, Entman ML (1992). Reactive oxygen in skeletal muscle. II. Extracellular release of free radicals. J Appl Physiol.

[33] Heacock AM, Kerley D, Gurda GT, Van Troostenberghe AT, Fisher SK (2004). Potentiation of the osmosensitive release of taurine and D-aspartate from SH-SY5Y neuroblastoma cells after activation of M3 muscarinic cholinergic receptors. J Pharmacol Exp Ther.

[34] Décombaz J, Reinhardt P, Anantharaman K, von Glutz G, Poortmans JR (1979). Biochemical changes in a 100 km run: free amino acids, urea, and creatinine. Eur J Appl Physiol Occup Physiol.

[35] Kavianipour M, Wikstrom G, Ronquist G, Waldenstrom A (2004). Validity of elevated interstitial levels of taurine as a predictor of myocardial ischemic injury. Amino Acids.

[36] Gambhir KK, Archer JA, Bradley CJ (1978). Characteristics of human erythrocyte insulin receptors. Diabetes.

[37] Gambhir KK, Agarwal VR (1991). Red blood cell insulin receptors in health and disease. Biochem Med Metab Biol.

[38] Sanchez-Margalet V, Valle M, Lobon JA, Maldonado A, Escobar F, Perez CR (1994). Diminished insulin receptors on erythrocyte ghosts in nonobese patients with essential hypertension independent of hyperinsulinemia. J Cardiovasc Pharmacol.

[39] Ronquist G, Rudolphi O, Engstrom I, Waldenstrom A (2001). Familial phosphofructokinase deficiency is associated with a disturbed calcium homeostasis in erythrocytes. J Intern Med.

[40] Branth S, Ronquist G, Stridsberg M, Hambraeus L, Kindgren E, Olsson R (2007). Development of abdominal fat and incipient metabolic syndrome in young healthy men exposed to long-term stress. Nutr Metab Cardiovasc Dis.

[41] Nehlsen-Cannarella SL, Fagoaga OR, Nieman DC, Henson DA, Butterworth DE, Schmitt RL (1997). Carbohydrate and the cytokine response to 2.5 h of running. J Appl Physiol.

[42] Steinacker JM, Lormes W, Reissnecker S, Liu Y (2004). New aspects of the hormone and cytokine response to training. Eur J Appl Physiol.

